# "How I do it: utilization of high-pressure sealants in aortic reconstruction"

**DOI:** 10.1186/1749-8090-4-27

**Published:** 2009-06-26

**Authors:** John A Elefteriades

**Affiliations:** 1 Cardiothoracic Surgery, Yale University School of Medicine, New Haven, Connecticut, USA

## Abstract

**Background:**

Suture-line hemostasis, reinforcement of friable tissue, and adhesion prevention are key concerns for patients undergoing cardiac surgery for aortic reconstruction. Failure to secure hemostasis at anastomotic junctures and reinforce fragile tissue may lead to increased blood loss, additional blood product requirements, increased operative time, and, in extreme cases, reoperation. Patients with aortic pathology may also be at higher risk for reoperation, and adhesion formation from prior surgery is an added risk at resternotomy. The advent of high-pressure sealants has been of benefit in helping to alleviate these perioperative challenges.

**Methods:**

The author utilizes two high-pressure sealants for aortic reconstructive procedures. The first is made of two polymers of polyethylene glycol (PEG) [Coseal^®^, Baxter Healthcare, Corporation], and is used to secure anastomotic suture-line hemostasis and for adhesion prevention. The second is a bovine serum albumin-glutaraldehyde (BSAG) glue [BioGlue^®^, CryroLife, Inc.], used for the repair of dissected aortic tissue and in reinforcing ("tanning") fragile aortic tissues. The techniques for application in select aortic reconstruction procedures are described.

**Results:**

To substantiate the hemostatic clinical benefit observed by the author, 60 consecutive major thoracic aortic operations in 57 patients in whom PEG sealant was used were retrospectively reviewed. Although comparisons with other agents were not performed for this descriptive report, bleeding results were very favorable for these types of operations. The strong clinical impression is that topical hemostatic application of PEG sealant to anastomotic suture lines is helpful in preventing bleeding.

**Conclusion:**

In major aortic reconstructive procedures the need for anastomotic sealing performance, reinforcement of friable tissues, and adhesion prevention should not be underrated. High-pressure surgical sealants represent an important surgical adjunct, and the author has found the use of both PEG sealant and BSAG glue advantageous in aortic reconstruction and repair.

## Background

*"Chance fights ever on the side of the prudent." *– Euripides

While mastery of the technical skills required to perform cardiac surgery may progress at a rapid pace, we are ever at the mercy of the coagulation cascade when seeking a successful operative outcome. Awareness of this truth has become especially poignant to cardiac surgeons following the removal of aprotinin from our armamentarium [1].

Repair and reconstruction of the aorta are major cardiac surgical procedures which often require implantation of prosthetic graft materials primarily composed of Dacron, which can be prone to recalcitrant bleeding at anastomotic sites [2,3]. The lability of the hemostatic situation is further exacerbated by intraoperative anticoagulation therapy required for cardiopulmonary bypass (CPB) and/or coagulation consequences of deep hypothermic circulatory arrest (DHCA) [4,5]. The friable, thin tissue often seen in aneurysm and connective disease patient populations, dissected aortic tissues, and porosities/tracks left by the larger needles required to suture thick aortic tissue may also be factors that contribute to increased blood loss in aortic reconstructive procedures. Failure to secure hemostasis at anastomotic junctures may lead to increased blood loss, additional blood product requirements, increased operative time and, in extreme cases, reoperation [2-4,6-8].

### High pressure sealants: Clinical uses and properties

#### Anastomotic suture-line hemostasis

**Figure 1:**
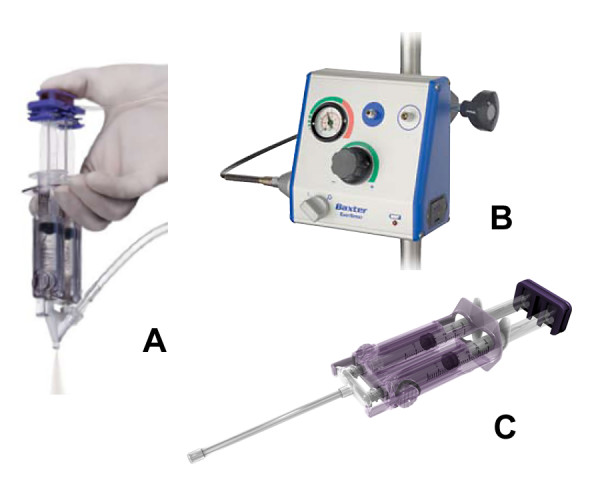
**A) Polyethylene glycol (PEG) surgical sealant (Coseal^®^) spray applicator**. B) "Easy Spray" regulator used with spray applicator. C) Self-contained standard applicator. (Photos courtesy of Baxter Healthcare Corporation).

**Figure 2:**
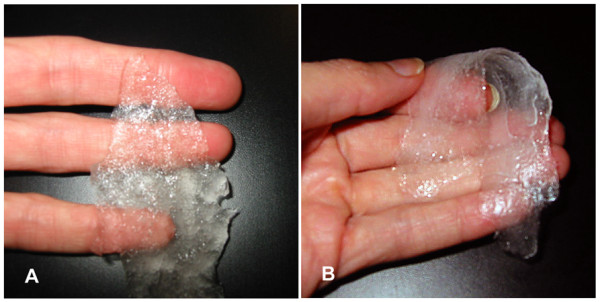
**A) PEG sealant following polymerization with semi-opaque hydrogel consistency**. B) Sealant is pliable, and does not restrict the movement of surrounding tissues. It maintains a secure seal even under the influence of high pressures exerted by the aorta.

The advent of high-pressure sealants has been of benefit in helping to alleviate the perioperative challenges described, especially when operating on the aorta [2,3,6,7,9,10]. Out of the many options available for securing hemostasis at the anastomotic suture-line, the author often utilizes a surgical sealant made of two polymers of polyethylene glycol (PEG) [Coseal^®^, Baxter Healthcare Corporation][10]. This sealant produces an adherent, nearly clear, gel-like pseudo-clot at the site of application, which requires no participation of the coagulation cascade whatsoever. Thus, this sealant can be effective even in the face of marked deficiencies in the patient's intrinsic coagulation mechanism. One may use either a spray device which requires a separate pump [Fig 1A, B], or opt for a self-contained standard applicator [Fig 1C]. The polymers gel within five seconds and set within 60 seconds. The result is a flexible, clear, degradable hydrogel that seals and adheres to tissues (even irregular anastomotic structures) to which it is applied [Fig 2][2,3,6,7,9,10].

The sealant remains flexible and does not restrict movement of surrounding structures; however, it maintains a tight seal through covalent tissue bonds even under the influence of high pressures in vessels such as the aorta (demonstrated to seal leak pressures of 660 ± 150 mmHg in porcine models) [2,3,6,7,9,10]. In a study conducted to compare the mechanical properties of surgical glues used in aortic root replacement, PEG sealant demonstrated hemostatic efficacy while remaining flexible and compliant. Thus, it allows for normal physiologic dilation without contributing to elevated wall stress by stiffening surrounding tissues which may weaken tissue structure and potentiate late pseudoaneurysm formation [11].

#### Repair of dissected aortic layers

Bovine serum albumin-glutaraldehyde (BSAG) glue [BioGlue^®^, CryroLife, Inc.] has adhesive as well as sealant properties. It is provided in a self-contained applicator with optional tip extenders [12]. BSAG glue begins to polymerize within 20 to 30 seconds, reaches its bonding strength within two minutes, and is very useful in repairing dissected aortic layers and in reinforcing ("tanning") fragile aortic tissues. The author is a strong advocate for the use of BSAG glue in the setting of acute aortic dissection, or in situations of severe tissue fragility, in which the tissue tanning and strengthening properties of the glutaraldehyde can be life-saving [4]. It has been reported as a means of securing suture-line hemostasis, however, this use is not recommended by the author as the potential for product leakage through needle-holes may lead to thrombosis or embolization [13,14]. Adverse events associated with the use of BSAG glue have also been reported and include phrenic nerve damage leading to diaphragmatic paralysis, false aneurysm, and local and distant complications from embolized particles released into the vessel lumen through needle holes and as a result of over-application of material [15-17].

#### Adhesion prevention

One key property of PEG sealant in cardiac surgery is that the hydrogel swells to 4× the initial volume within 24 hours of application, allowing it to act effectively as a mechanical barrier to cellular infiltration keeping tissues separated while the inflammatory process subsides. By separating tissues during the healing process, PEG sealant has been shown to be effective at preventing surgical adhesions in both cardiac and gynecologic surgery [18-20]. This is an especially important consideration in patients in whom reoperation is anticipated (pediatric patients, younger adult patients, etc.) or expected (when placing left ventricular assist systems), as the potential for formation of dense and tenacious adhesions makes resternotomy difficult and potentiates injury to structures such as the aorta, right ventricle, right atrium, innominate vein, innominate artery, aortocoronary grafts, etc. [21]. Adhesions may also potentially constrict the heart, potentiate right ventricular dysfunction, and/or be a factor in the promotion of graft occlusion [22-25]. Clinicians should be aware that PEG sealant is approved for the indication of adhesion prevention in Europe, however, approval for use in this indication has not yet been granted in the United States.

The optimal duration for which early adhesions must be prevented to reduce longer term scar formation is unknown; however, evidence suggests that even 6 to 12 hours may be sufficient [26,27]. The PEG sealant swells to 4× the initial volume within 24 hours of application, allowing it to act effectively as a barrier to cellular infiltration. However, one must be careful to apply only a thin, uniform layer of sealant as thicker applications do not increase benefit and may lead to mass effects or mechanical compression complications [28].

#### Biocompatibility and biodegradability

PEG sealant and BSAG glue contain reactive substances to adequately perform their intended function. Both products adhere to tissues by covalent chemical bonds [29]. Thus, a chemical reaction takes place between the product and the tissue surface, and a foreign-body reaction to the implanted material is inevitable. The degree of the response is related to many factors including implantation site, reactivity of the patient, and could possibly be related to the type and amount of product used. PEG sealant is synthetically-derived, and there are no human or bovine blood-based additives. It is therefore, highly biocompatible and the observed reactivity has been considered minimal [30]. BSAG glue contains a small amount of bovine additive, however, reactivity seems to be related to the release of glutaraldehyde. One study indicated that polymerized BSAG glue may induce cytotoxic effects both in vitro and in vivo, and recommends that it be restricted to the aortic dissection procedure as other tissues are sensitive to the glutaraldehyde released [31].

Biodegradability of surgical sealants is a critical factor and directly relates to the long-term effects. PEG sealant and BSAG glue both degrade following implantation, however, the rates and mechanisms by which this occurs are very different [29]. The resorption rate of PEG sealant is fairly rapid. In an in vitro study, hydrolytic degradation was demonstrated after several days of incubation at 37°C [30]. In vivo, PEG sealant degraded within < 30 days of implantation and could not be discerned grossly or histologically at 30 days [7]. Because the major component of BSAG glue is albumin, it degrades via proteolysis and is resorbed slowly [29]. Histologically, the material was detectable after 1 year in a goat model [32].

## Methods

### General application techniques

**Figure 3:**
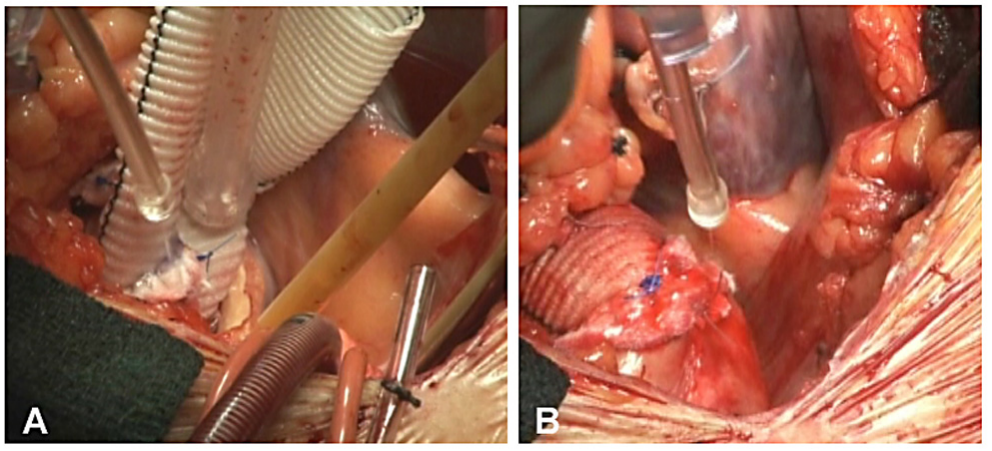
**A): Intraoperative view of PEG sealant coverage of proximal and B) distal aortic anastomoses utilizing a self-contained, standard applicator**.

For PEG sealant and BSAG glue, the suture line must be absolutely dry before application. Careful drying with sponges usually suffices. If necessary, sponge drying may be supplemented by insufflations of CO2 just before sealant application. When using PEG sealant, the spray applicator may be used to dry the surface by covering the hole at the upper end without depressing plunger (to extrude sealant). A thin, uniform layer of sealant is optimal [Fig 3]. Excessive sealant application will not increase benefit and may lead to adverse effects.

To insure uniform coverage, the treatment site may be rotated to facilitate exposure of all surfaces and/or the tip of the applicator may be bent. The sealant should be applied in one continuous motion for thorough, even coverage, moving quickly rather than pausing, which may cause the tip to clog. Extra applicator tips should be made available for rapid replacement should clogging occur. Following polymerization, PEG sealant remains pliable. If required, reinforcement sutures at the anastomic suture line can be placed through it, though this is seldom necessary. One should remember that surgical sealants and adhesives are not a replacement for meticulous surgical technique and, in spite of rapid polymerization, an inadequate repair may allow for entry of the sealant into the vessel lumen.

### Ascending aortic reconstruction

There are several disorders that necessitate reconstruction in both the ascending aorta and aortic arch. The most common are ascending aneurysm, ascending dissection, arch aneurysm, and descending pathology requiring formal aortic arch replacement. The most serious complications of aortic aneurysm are aortic dissection and rupture which are inherently lethal conditions. The most important concern should be patient survival [33]. If the patient survives the acute episode this constitutes a success, regardless of later onset of further aortic problems. The early mortality for urgent operation for acute aortic dissection ranges from 15% to 25%, depending largely on institutional experience [4,33]. While such results represent dramatic improvement from earlier eras, surgical science still has room for progress in the treatment of this challenging disorder. One of the critical elements to achieving patient survival is complete hemostasis.

#### Supracoronary tube and composite graft reconstruction

The author's preferred technique for operations on the ascending aorta is via two-stage atrial/femoral artery cannulation and perfusion, arresting the heart through antegrade and retrograde cardioplegia, cross-clamping the aorta, and performing the proximal anastomosis. The aorta is then unclamped under deep hypothermic circulatory arrest (DHCA) and the operation proceeds with the distal anastomosis. Performing the proximal anastomosis first is preferred by the author, as the required exposure deep in the aortic root is more difficult after the distal graft is attached. Perfusion is then resumed using the original femoral cannula. The author has previously published on the safety of resuming perfusion through this route even in aortic dissection [33,34]. In the minority of patients with descending atheroma (unusual in this patient group) axillary cannulation is used.

Prior to application of the surgical sealant, which is the final step in securing the anastomotic suture-line, the author also recommends reinforcement of posterior anastomotic suture lines (both in a tube graft, distal in a composite graft), with interrupted, single-pledgeted sutures as this area becomes inaccessible following anastomotic closure. Although it does take a bit of extra time, the author considers these minutes well-spent in terms of the added strength provided to the arterial closure. A Teflon felt strip is then applied circumferentially as the anastomosis proceeds with 3-0 Prolene sutures. MH needles are recommended to adequately penetrate dense aortic tissue and facilitate grasping. Finally, in the case of composite graft reconstruction, placement of felt washers to secure the coronary buttons is recommended [35].

#### Repair of dissected aortic layers

For an acute ascending aortic dissection, the separated layers of the aortic wall (the "intima" and the "adventitia") are re-approximated prior to anastomosis to the prosthetic graft via a "sandwich" technique of Teflon strips placed inside and outside the vessel wall secured by radially-oriented horizontal mattress sutures. The felt adds considerably to the strength of the reconstituted aortic wall. As previously discussed, the repair may also be supplemented with BSAG glue used sparingly to produce adherence between layers and to strengthen the fragile tissues through its tanning properties.

#### Sealing of the proximal anastomosis

Following proximal anastomotic completion of a composite graft, the PEG surgical sealant is applied via a spray or standard applicator circumferentially (360°) to the anastomotic juncture and coronary buttons [see Additional file 1]. Sealing is performed immediately following completion of the proximal anastomosis, as this area will disappear from view after the heart is reperfused [see Fig 3A]. To insure that a thin, uniform layer of sealant is applied to the anastomotic site, the graft may need to be carefully shifted and the applicator rotated circumferentially. Keep in mind that thicker applications of sealant will not increase benefit, and may lead to longer polymerization time and/or mass effect complications due to anticipated swelling of product up to 4× its volume [28]. As the distal portion of the graft remains open at this stage in the operation, one should take care (especially when using the spray applicator) not to allow product to enter the vessel lumen.

#### Sealing of the distal anastomosis

Following completion and sealing of the proximal aortic anastomosis, the distal anastomosis is performed in like fashion with posterior pledgets, a circumferential Teflon strip, and PEG sealant [see Fig. 3B]. By reaching around the graft and gently mobilizing it, a near 360° application can be achieved. If necessary, the applicator tip may be bent to provide coverage in hard-to-reach areas.

If product is still remaining in the applicator following the proximal anastomosis, it may be used for the distal anastomosis. Depending on the coverage area, one should choose the appropriate amount to have mixed and ready for sealing. When using a spray tip, application may be easily resumed. When using a self-contained applicator, a tip replacement may be required, as the tip tends to clog following a pause in the procedure.

#### Simultaneous sealing of the proximal and distal anastomoses

In some instances, as for a simple tube graft, it may be appropriate to seal both the proximal and distal anastomoses simultaneously following completion of the distal anastomosis. The author sometimes prefers to reperfuse the aorta following removal from DHCA, and complete rewarming and weaning from bypass, thus allowing native hemostatic mechanisms to act. Subsequently the area is dried with sponges in anticipation of sealant application, which is done just before chest closure. This sequence is appropriate for anastomoses that are still well exposed after the heart is filled and bypass is discontinued. For patients with reoperative potential, an added benefit may be derived from utilizing the spray applicator within the chest cavity/surgical site for adhesion prevention as previously described.

### Aortic arch replacement

The author frequently modifies the technique for arch replacement from the standard method. This is accomplished by confining the Carrel patch to the innominate and left carotid arteries. This makes for a more manageable patch, located close to the operator and fully accessible for subsequent hemostasis. This factor of accessibility becomes even more important in the case of acute dissection of the aortic arch, as the dissected aorta is friable, and bleeding can frequently ensue, requiring tension-free exposure and placement of additional sealant and sutures if necessary. The smaller two-vessel procedure meets all of these requirements for accessibility. Also, this smaller patch can be anastomosed in relatively short order, thus decreasing the length of cerebral ischemia and DHCA. After the patient is weaned from bypass, a side-arm graft to the subclavian artery is placed. In the case of patient instability, the subclavian artery can be simply ligated; ischemic problems would be rare with such a proximal ligation of the subclavian artery as there are extensive collaterals via the thyrocervial trunk, internal mammary artery, and other routes.

PEG sealant is applied to the Carrel patch and to all anastomotic suture-lines. As mentioned previously, reinforcement sutures may still be placed following application and polymerization of the product if one notes that suture-line hemostasis has not been secured following reperfusion.

## Results

Cardiac surgeons have endeavored to identify new protocols and adjunctive methodologies for reducing perioperative blood loss and the use of exogenous blood products, especially in high-risk procedures. To substantiate that PEG sealant has been of benefit in this regard, the experience of 57 patients (41 male/16 female) patients were examined [Tables 1, 2]. We reviewed 60 consecutive major thoracic aortic operations in patients who received PEG sealant from June through December of 2008 (following the removal of aprotinin). In the majority of patients, PEG sealant was used in conjunction with aminocaproic acid [Amicar^®^, Xanodyne Pharmaceuticals, Inc.].

**Table 1:** Demographic and perioperative data for patients receiving PEG sealant

**Variables**	**Data**
**Total Patients**	57

**Total Operations**	60

**Total Reoperations (2 in same patient)**	3 (5%)

**Gender**	

Male	41 (72%)

Female	16 (28%)

**Age (years)**	

Mean	58

Range	26–89

**Weight (kilograms)**	

Mean	91

Range	50–147

**pRBC Transfusion Requirements (mean units)**	

Intraoperative	0.8

Postoperative	0.7

Procedures without pRBC transfusion	28 (47%)

CPB Time (minutes)*	

Mean	137

Range	27–210

Aortic Cross-Clamp (minutes)*	

Mean	106

Range	25–154

DHCA Time (minutes)*	

Mean	31

Range	19–52

Abbreviations: pRBC: packed red blood cells; CPB: cardiopulmonary bypass; DHCA: deep hypothermic circulatory arrest.

*Mean/range calculated for those procedures in which CPB, DHCA, and/or aortic cross-clamp.

**Table 2:** Procedural details for patients receiving PEG sealant

**Procedure/Reconstruction**	**Number of Patients (n = 57)**
Aortic valve replacement/aortoplasty	10 (17%)

Root-sparing ascending aorta	5 (8%)

Composite ascending aorta	17 (28%)

Root-sparing ascending aorta/hemiarch	6 (10%)

Composite ascending aorta/hemiarch	4 (7%)

Root-sparing ascending aorta/arch	6 (10%)

Composite ascending aorta/arch	2 (3%)

Descending aorta/reconstruction	2 (3%)

Other	8 (14%)


**Total Operations**	**60**

Note 1: Six (6) procedures involved associated CABG.

Note 2: Three (3) procedures (other) were reoperations for bleeding.

Although comparisons with other agents were not performed for this descriptive report, the author feels that bleeding results were very favorable for these types of operations. The strong clinical impression is that topical hemostatic application of PEG sealant to anastomotic suture lines is helpful in preventing bleeding, especially in the current post-aprotinin era.

## Discussion

Although a number of choices are available for suture-line hemostasis and repair of dissected aortic tissue, each has specific advantages and liabilities. PEG sealant and BSAG glue are fundamentally different than traditional hemostatic interventions because they are independent of the blood coagulation cascade. This property makes them ideal for cardiac surgery because they remain effective even in patients with severe coagulation deficiencies due to preoperative exposure to prescribed anticoagulants and herbal/vitamin supplements as well as intraoperative heparinization for CPB and use of DHCA.

In addition to the many challenges already discussed in terms of securing hemostasis at anastomotic suture lines in aortic reconstruction, there has been a steady increase in the use of herbal and vitamin supplements that interfere with the coagulation process. The most notable are ginseng, gingko biloba, and omega-3 fatty acids [36-40]. The estimated prevalence of dietary-supplement use among US adults was 73% in 2002 and this rate continues to increase annually [36]. Many patients do not consider the use of herbal and vitamin supplements to be relevant to medical practice. Consequently, they may not tell their clinicians about use of these substances [40]. Thus, it is prudent for clinicians to routinely identify which supplements their patients may be using.

In addition to herbal and vitamin supplements, the widespread use of short or long-term anticoagulation therapy (e.g., clopidogrel, warfarin, aspirin, etc.) has also led to challenges in securing hemostasis in cardiac surgical procedures [41,42]. With recommended loading doses of clopidogrel as high as 900 mg in select patients [43], operating on patients on an emergent or elective basis in whom clopidogrel and other anticoagulant therapies are not discontinued in advance of surgery is a major cause for concern. These medications make coagulation in any surgical patient quite tenuous. In the case of clopidogrel, a recent study indicated that surgical patients taking this drug within 3 days of their procedure required almost twice as many reoperations [42].

In addition to suture-line hemostasis, PEG sealant has been shown to be effective at preventing adhesion formation in cardiac and gynecologic surgery [18-20]. However, although PEG sealant has been studied and is licensed for adhesion prevention in Europe, it has not yet been licensed for this indication in the United States, so clinicians should evaluate use in this capacity carefully.

## Conclusion

The best outcome, in terms of appropriate hemostasis in cardiac surgery, resides in prevention and prudence. In major aortic reconstructive procedures the need for anastomotic sealing performance, adhesion prevention, and reinforcement of friable tissues should not be underrated. High-pressure surgical sealants represent an important surgical adjunct, and the author has found the use of both PEG sealant and BSAG glue advantageous in aortic reconstruction and repair.

## Competing interests

The author declares that they have no competing interests.

## Authors' contributions

The manuscript development and techniques described herein were performed by the author.

## Supplementary Material

Additional file 1 **Intraoperative demonstration of PEG sealant application**. Techniques for intraoperative application of PEG sealant at aortic anastomotic sites are demonstrated and described. Click here for file
